# The Burden of Serious Fungal Infections in Cameroon

**DOI:** 10.3390/jof4020044

**Published:** 2018-03-30

**Authors:** Christine E. Mandengue, David W. Denning

**Affiliations:** 1 Department of Internal Medicine (Dermatology), Université des Montagnes, Bangangté P.O. Box 208, Cameroon (Central Africa); 2 National Aspergillosis Centre, Wythenshawe Hospital and The University of Manchester, Manchester M13 9PL, UK; ddenning@manchester.ac.uk

**Keywords:** HIV/AIDS, fungal infections, pulmonary infections, opportunistic infections, Cameroon

## Abstract

Fungal infections are frequent in Cameroon, and invasive fungal infections are sometimes detected, usually in HIV-infected patients. For these reasons, we have estimated the burden of fungal infections. Using published literature and population estimates for the at-risk group, we used deterministic modelling to derive national incidence and prevalence estimates for the most serious fungal diseases. HIV infection is common and an estimated 120,000 have CD4 counts <200 × 10^6^/mL and commonly present with opportunistic infection. Oesophageal candidiasis in HIV is common, and in poorly controlled diabetics. We estimate 6720 cases of cryptococcal meningitis, 9000 of *Pneumocystis* pneumonia, 1800 of disseminated histoplasmosis annually complicating AIDS, and 1200 deaths from invasive aspergillosis in AIDS, but there are no data. We found that 2.4% of adults have chronic obstructive pulmonary disease (COPD) and 2.65% have asthma, with “fungal asthma” affecting 20,000. Chronic pulmonary aspergillosis probably affects about 5000 people, predominantly after tuberculosis but also with COPD and other lung diseases. Also, tinea capitis in schoolchildren is frequent. Overall, an estimated 1,236,332 people are affected by a serious fungal infection. There is an urgent need for government and clinician attention, improved laboratory facilities, fungal diagnostic tests, and competent laboratory technicians, as well as all World Health Organization (WHO)-endorsed essential antifungal drugs to be made available, as only fluconazole is registered and available in the country.

## 1. Introduction

Cameroon is a tropical Central African country located in the Gulf of Guinea. As in many tropical countries, infectious diseases are common and have particularly increased with the HIV epidemic, which is a huge problem in Cameroon. Although opportunistic infections occur frequently in AIDS persons, tuberculosis (TB) is the Government’s main public health concern [1]. Yet, there were nearly 30,000 estimated deaths from AIDS in 2016, a figure unchanged since 2012 and greater than in 2001. The narrow focus on TB has prevented any significant developments with respect to the other major causes of death in AIDS, namely fungal diseases, and in addition, data on the national incidence and prevalence of fungal infections are unavailable.

Limited studies and some unpublished studies record frequencies of mild superficial mycosis [2,3,4,5] or life-threatening invasive fungal infections in the HIV-infected population in Cameroon [6,7,8,9,10,11,12]. Ignorance of serious fungal infections, lack of awareness or involvement of practicing clinicians and the government, together with limited technical and structural health capabilities in Cameroon, have meant there is no register for any fungal disease. This lack of surveillance data combined with the unavailability of any non-culture diagnostic test for fungi, other than microscopy, reinforces a sense that these infections are rare or non-existent. Given that such infections are perceived to be rare, there is no call for any antifungals other than fluconazole to be made available in the country—a negative reinforcement of the absence of a problem in Cameroon.

For these reasons, here we have estimated the burden of serious fungal infections in Cameroon in the hope that highlighting the enormous gap between what is the current status quo and the likely actual situation will stimulate research, capacity development, and improved care [13].

## 2. Materials and Methods

In this study, we searched publications on literature in order to identify epidemiology papers reporting fungal infections frequencies from Cameroon, using PubMed. The terms used were “fungal infections in Africa”, “fungal infections in Cameroon”, and “opportunistic infections in Cameroon”.

We used the population for the at-risk group and deterministic modelling to derive national incidence and prevalence estimates for the most serious fungal diseases when data was unavailable.

We sourced the total Cameroonian population from the National Statistical Institute 2017 reports [14]. HIV/AIDS and tuberculosis prevalence were sourced from the World Health Organization (WHO) 2016 reports [15] and the Ministry of Public Health of Cameroon 2014 reports [16]. The assumptions made in estimating burden are shown in Table 1, with the pertinent references.

## 3. Results

### 3.1. Country Profile

Currently, the Cameroonian population is estimated at ~24.23 million, of whom 43.6% are aged <15 years old and 50.1% are females [14]. There are an estimated 613,731 persons living with HIV; 555,120 adults aged between 20 and 44, and 58,611 children aged <14 years old. In 2016, annual deaths from AIDS was ~29,327, and ~120,000 persons had CD4 counts <200 × 10^6^/mL, at risk of presenting with opportunistic infections and ~240,000 not receiving (free) antiretroviral therapy (ART) [15,16]. The gross domestic product was $1033 per capita in 2016.

### 3.2. Serious Fungal Infections

Table 2 shows our estimates of the most serious fungal infections in Cameroon. We have not added sensitivity analyses, because the basis for the estimates themselves is mostly inferred from data from other countries, and more precise local estimates are desirable before more sophisticated modeling is done.

#### 3.2.1. Pulmonary Conditions and Infections

In 2015, ~25,975 people were notified as infected by TB, 16,000 of them also with HIV-infection and 54% of these in receipt of free ART [15]. We assumed that only those with pulmonary TB who survived 12 months would develop chronic pulmonary aspergillosis, an estimated 19,762 people. The prevalence of chronic obstructive pulmonary disease (COPD) was 2.4% in adults (over 19) based on published studies in Yaoundé in HIV-positive and -negative adults [30,31], and at risk of chronic pulmonary aspergillosis. It was estimated that 25% of COPD patients are admitted to hospital per year and are at risk of invasive aspergillosis. The estimated number of adults with clinical asthma is 385,260 (2.65%) [31], and 2.5% of asthmatics develop allergic bronchopulmonary asthma (ABPA). Assuming that about 10% of adults have severe asthma (38,526), we assumed that severe asthma with fungal sensitization (SAFS) was present in 33% of these patients. ABPA and SAFS together are called “fungal asthma”, and over 20,000 adults suffer from this. Cystic fibrosis has not been not reported in Cameroon.

Concerning aspergillosis, estimates of the annual incidence of invasive aspergillosis (IA) were made [32]. About 1175 IA cases (leukaemia, lymphoma, and COPD) were anticipated, not including those complicating lung cancer or HIV. As over 4% of patients with AIDS who die are found to have IA at autopsy (consistently in Italy over 18 years) [33], it is possible that another 1200 IA cases occur each year, but data are lacking from Africa and so are not included.

Chronic pulmonary aspergillosis (CPA) annual incidence was estimated as previously (1265 cases) with a 5-year-period prevalence of 4983 (assuming 80% of cases occur after TB) [23]. These estimates are broadly (qualitatively) supported by a recent cross-sectional study from Nigeria [24]. A recent Cameroonian study in Yaoundé recorded 20 cases of complex pulmonary aspergilloma in three years (2012–2015) in immunocompetent patients with a past history of TB, which were treated surgically, confirming that CPA occurs in Cameroon [25]. In addition, 8844 patients with ABPA complicating asthma in adults were expected and 11,675 cases of SAFS. Other less severe forms of aspergillosis were not estimated, including rhinosinusitis, onychomycosis [34], or otitis externa.

#### 3.2.2. Opportunistic Fungal Infections Complicating HIV Infection

Opportunistic fungal infections are common in HIV-infected persons in Cameroon, but are not well documented. *Pneumocystis* pneumonia (PCP) was estimated at an annual rate of 7.5% in those with CD4 counts <200 × 10^6^/mL. Although national data is unavailable, reports of high frequency in cross-sectional studies attest to endemicity in Cameroon. Nkinin and colleagues reported 82% positivity to *P. jirovecii* antibody using ELISA on 349 randomly collected serum samples in 2009 (50% from HIV positive people) [6]; Riebold and Enoh found that 31.6% of patients in 2014 were colonized with *P. jirovecii* by PCR detection of induced sputum, most of them being HIV-infected [7]. Our estimate could be an underestimate therefore.

Cryptococcal meningitis (CM) was estimated to occur at a rate of 11% over 2 years in patients with CD4 counts <200 × 10^6^/mL (6720 cases). Reports from 2012 to 2015 showed various prevalence figures from 9.9% to 28.1% in slightly different HIV populations [8,9,10]. Our annual incidence estimate is higher than that of Rajasingham et al., (*n* = 3602), which was based on a prevalence of 6.1% of cryptococcal antigenaemia in those with CD4 counts <100 × 10^6^/mL [35].

Disseminated histoplasmosis (DH) was estimated to occur at an annual rate of 1.5% in HIV patients with CD4 counts <200 × 10^6^/mL, which means that ~1800 patients probably have this life-threatening, and probably fatal, infection each year. National data on this fungal infection is unavailable. However, a Cameroonian study reported in 2015 a prevalence of 13% of DH due to *Histoplasma capsulatum* var. *capsulatum* (Hcc) in HIV-infected persons [11]. The same author previously detected three cases of DH in HIV-infected persons: the first case was due to *Histoplasma capsulatum* var. *duboisii* (Hcd), successfully treated with boluses of fluconazole, the two other patients being Hcc histoplasmosis and detected post-mortem on skin biopsy and on peripheral blood smear [36,37]. Nonetheless, a recent publication recorded a total of 15 cases of both Hcc (9/15) and Hcd (6/15) histoplasmosis in Cameroon, HIV-positive persons being more numerous (12/15) than negative patients (3/15) [38].

Oesophageal candidiasis is a frequent problem in HIV patients and was estimated to occur in at least 43,300 people each year (177/100,000). More common is oropharyngeal candidiasis, estimated in 108,000 HIV-infected patients. In addition to this large number of cases, a local report from 2015 found 23.5% of poorly controlled diabetics to have oropharyngeal candidiasis [39]. We do not have a good denominator for diabetes in Cameroon, or what proportion are poorly controlled.

#### 3.2.3. Other Fungal Infections

Data was not found on candidaemia, candida peritonitis, or other forms of invasive candidiasis, and so we have estimated annual incidences of 5/100,000 and 0.75/100,000, based on other countries [18,19]. Likewise, we did not find any report on recurrent vulvovaginal candidiasis from Cameroon, although it is present, and so we used a 6% proportion of women aged between 5 and 50 as a prevalence estimate. This translates to nearly 350,000 women (2845/100,000) mostly without any underlying disease.

Fungal keratitis was not recorded.

Mucormycosis was estimated 0.2/100,000. Only five cases (one basidiobolomycosis) were reported in 1992 [40]. Data on chromoblastomycosis and mycetoma was not found.

Tinea capitis in schoolchildren is possibly the largest burden of significant superficial fungal infections, with frequencies from 31 to 66% [2,3,4]. Our estimate is that ~721,000 children have tinea capitis, a rate of 3240/100,000. Onychomycosis was also found at high rate (51%) especially in diabetic patients [5].

## 4. Discussion

In this study, we attempt to highlight the burden of serious fungal infections in Cameroon.

Aspergillosis is poorly reported and infrequently diagnosed in this country. Assuming the endemicity of TB, which is considered as the main opportunistic AIDS-defining infection and the main cause of death in AIDS persons in Cameroon [41], high frequencies of post-TB aspergillosis are expected as in other endemic sub-Saharan African countries for TB [42,43,44,45,46]. Surprisingly, only one Cameroonian publication recording 20 cases of complex pulmonary aspergilloma in immunocompetent patients has been published [25]. This contrasts with our estimate of nearly 5000 cases. No other forms of aspergillosis were reported, including invasive aspergillosis. The rarity of publications may be explained by ignorance or lack of awareness of practicians who could misdiagnose aspergillosis as TB given the similarities of clinical symptoms (cough and bloody sputum, dyspnea, and weight loss) and on thoracic imaging (nodules, cavities, and infiltrates), leading probably to presumptive antituberculosis treatment. Fever is uncommon with aspergillosis but more common with TB. The lack of efficient laboratory methods for diagnosis of aspergillosis (microscopy or culture on tissue biopsy, or IgG antibody response to *Aspergillus* spp.) is also a major factor in under-diagnosis. Moreover, asymptomatic patients are never encountered in sub-Saharan African countries, patients attending hospital only when they feel their lives are threatened. Many patients thus die without a definite diagnosis of aspergillosis. As no antifungals are available in Cameroon that are effective for aspergillosis (itraconazole, voriconazole, amphotericin B, or echinocandins), we must estimate that all those with invasive disease die and most of those with CPA also die.

Histoplasmosis is underreported in Cameroon. This may be explained by ignorance or lack of awareness or involvement of medical practitioners, almost no laboratory diagnostic facilities other than microscopy, and no qualified laboratory technicians. However, both Hcc and Hcd histoplasmosis coexist in Cameroon as in other sub-Saharan African countries, occurring either in HIV-infected or -negative patients, with various clinical presentations but presumably a fatal outcome in patients with deep immunosuppression especially due to HIV infection [38,47,48]. It is therefore noteworthy that sub-Saharan African clinicians should think of histoplasmosis in case of a sudden occurrence of poor general condition with CD4 T cells count <100/mm^3^ in HIV-infected patients [49]; or in a patient suspected of tuberculosis with negative sputum on bacteriology examination, and treated with effective antituberculosis drugs without clinical improvement [36,50]; or in case of a prolonged fever occurring in a HIV+ person or a person of unknown HIV serology status [37,51,52,53,54]. Unfortunately, fluconazole (which is the only available antifungal treatment) is a sub-optimal therapy for disseminated (and other forms) histoplasmosis, itraconazole and amphotericin being more effective (but unavailable in this country).

CM is frequently reported with high frequencies in HIV-infected patients [9,10,12], given the cheap routine means of diagnosis (direct detection with India ink and culture equipment). Meanwhile, its real national prevalence is unknown as a consequence of a lack of research, out-of-pocket payments that prevents patients from consulting at hospitals, lack of any infection-monitoring program, and routine treatment of AIDS patients with fluconazole. Our estimate is two-fold higher than another recent estimate [12]. This needs further work to clarify.

As in parts of sub-Saharan African countries, the prevalence of PCP is unknown in the general population or underestimated, owing to the high rate of death in AIDS persons and in children aged <1 year, the lack of suitable diagnostics practices, and commonly administered cotrimoxazole prophylaxis in HIV-infected persons [27,28]. Not all PCP cases are “classical” and many have co-infections, so it is highly likely that many hospitalized AIDS patients have PCP that is not diagnosed or empirically treated.

Superficial fungal infections in Cameroon are dominated by an extraordinarily high prevalence of tinea capitis, especially in school-age children, as in other sub-Saharan African countries [2,3,4,55]. Even so, it is still underestimated as the diagnosis is usually clinically documented, mycology examination being reserved only for research studies. So, many cases of scaly scalp in infants or in adults, and many girls with braided hair are not accounted for, while they could have been detected as genuine (and infectious) cases on mycology examination [2,3].

Oropharyngeal candidiasis occurs commonly in patients with advanced HIV infection or other immunosuppressive conditions. However, its prevalence cannot be established with certainty in the whole population in Cameroon, given the lack of data. Oesophageal candidiasis is probably common but not often definitively diagnosed as there is a lack of endoscopy. Estimates for other serious fungal infections including candida peritonitis and candidaemia are certainly preliminary and we are unable to make any estimate of fungal keratitis, chromoblastomycosis, or mycetoma.

## 5. Conclusions

Although fungal infections are frequent in Cameroon, it is currently not possible to estimate their frequencies with certainty in the absence of good diagnostics, registers reporting well-documented data or prospective surveys. Practitioners should be involved and more aware of fungal infections in general and particularly on health-threatening diseases, in order to provide good clinical care. They urgently need government attention, improved laboratory facilities, fungal diagnostic tests, and competent laboratory technicians, as well as all the WHO-endorsed essential antifungal drugs to be made available, as only fluconazole is registered and available in the country.

## Figures and Tables

**Table 1:** Assumptions made in assessing burden.

Disease	Underlying Disease(s)	Incidence/Prevalence Used to Estimate Burden	Comments	Reference
Oesophageal candidiasis	HIV/AIDS	22% of patients with CD4 counts <200 × 10^6^/mL and 5% of ARV-treated patients		[17]
Candidaemia	Multiple hospitalized patients	5/100,000 population, 33% occurring in intensive care	Few patients managed in ICU currently.	[18]
Candida peritonitis	Major abdominal surgery, pancreatitis	50% of the ICU population with candidaemia		[19]
Recurrent vaginal candidiasis (>4×/year)	Pre-menopausal women	6% prevalence	Based on mean self-reported disease in Europe and US, reduced by 33% because of incorrect diagnosis.	[20]
Allergic bronchopulmonary aspergillosis	Asthma	2.5% of adults with asthma	Rare in children	[21]
Severe asthma with fungal sensitisation	Severe asthma	33% of the 10% of the most severe adult asthmatics	Uncommon in children. Fungal sensitization prevalence not known for Cameroon.	[22]
Chronic pulmonary aspergillosis	Tuberculosis (TB), COPD, prior pneumothorax, asthma, lung surgery	22% of patients with a cavity of pulmonary TB survivors (22%), 2% of those without a cavity. Patients with other pulmonary conditions contribute an additional 25% of cases		[23,24,25]
Invasive aspergillosis	Leukaemia, lymphoma, COPD	10% of acute myeloid leukaemia, an equal number of cases in all other haematological conditions + 1.3% of patients with COPD admitted to hospital	Patients with other conditions not included, including HIV/AIDS.	[26]
Cryptococcal meningitis	HIV/AIDS	11% over 2 years in patients with CD4 counts <200 × 10^6^/mL	The annual incidence rises as the CD4 count falls	[8,9,10]
*Pneumocystis* pneumonia	HIV/AIDS	15% over 2 years in patients with CD4 counts <200 × 10^6^/mL	Common in children but not estimated separately. Cases in non-AIDS cases not estimated.	[27,28]
Histoplasmosis	HIV/AIDS	1.5% over 2 years in patients with CD4 counts <200 × 10^6^/mL		[29]
Tinea capitis	Children 1–15 years	8.1%	The most conservative estimate is 8.1% in school aged children. There will be a slight over-estimate by including babies under 2 years.	[2]

**Table 2:** Estimates of most severe fungal infections in Cameroon.

Infection	Number of Infections per Underlying Disorder per Year					Rate/100 K	Total Burden
	None	HIV/AIDS	Respiratory	Cancer/Tx	ICU *		
Oesophageal candidiasis	-	43,300	-	?	-	193	43,300
Candidaemia	-	-	-	779	334	5.0	1113
*Candida* peritonitis	-	-	-	-	167	0.75	167
Recurrent vaginal candidiasis (4×/year +)	316,555	-	-	-	-	2845	316,555
ABPA *	-	-	8844	-	-	40	8844
SAFS *	-	-	11,675	-	-	52	11,675
Chronic pulmonary aspergillosis	-	-	4983	-	-	22	4983
Invasive aspergillosis	-	-	-	134	1041	5.3	1175
Cryptococcal meningitis	?	6720	-	?	-	30	6720
*Pneumocystis* pneumonia	-	9000	-	?	-	40	9000
Histoplasmosis	?	1800	?	?	?	16	1800
Tinea capitis	721,000	-	-	-	-	3240	721,000
Total burden estimated	1,037,555	60,820	25,502	913	1542	-	1,126,332

ICU = intensive care unit; ABPA = allergic bronchopulmonary aspergillosis; SAFS = severe asthma with fungal sensitization; * collectively called “fungal asthma”; + indicates rate per 100,000 females; ? indicates no reliable estimate possible.
